# Growth, physiological, antioxidants, and immune response of heteroclarias catfish fingerlings, to dietary *Moringa oleifera* leaves extract and its susceptibility to *Aeromonas hydrophila* infection

**DOI:** 10.3389/fphys.2026.1877528

**Published:** 2026-07-30

**Authors:** Bunmi E. Ajala, Al-ameen O. Abdulkareem, Thais P. da Cruz, Eijaz Ahmed Bhat, Ashraf M. Ahmed

**Affiliations:** 1 Department of Fisheries and Aquaculture Management, Federal University of Agriculture and Technology, Okeho, Nigeria; 2 Department of Aquaculture and Fisheries, University of Ilorin, Ilorin, Nigeria; 3 Department of Animal Science, Federal of Parana, Curitiba, Brazil; 4 Microbial Biochemistry Group, Institute of Phototrophic Microbiology, Heinrich-Heine University Düsseldorf, Universitätsstraße, Düsseldorf, Germany; 5 Department of Zoology, College of Science, King Saud University, Riyadh, Saudi Arabia

**Keywords:** blood profiles, fish, immune response, plant extract, production performance

## Abstract

*Moringa oleifera* leaves are an important phytobiotic agent due to their growth-promoting, anti-inflammatory, hepatoprotective, antimicrobial, and antioxidant properties. Thus, it has become a spicy and disease-control agent in traditional medicine. In this study, *Moringa oleifera* leaf extract (MOLE) was fed to hybrid catfish (Heteroclarias, *Clarias gariepinus ♀ × Heterobranchus longifilis ♂*) to evaluate its effects on growth, hematological parameters, liver enzyme activity, antioxidant capacity, immunity and susceptibility to *Aeromonas hydrophila* infection. Fish (n = 300 fish, mean weight 9.3 ± 0.2 g) were fed diets supplemented with 0.0, 0.5, 1.0, 1.5, or 2.0 g MOLE/kg diet for 56 days. Thereafter, fish were challenged with molecularly characterized *A. hydrophila* (ATCC 4356; 5.0 × 10^5^ CFU/mL) infection for 14 days. Growth performance and feed intake of heteroclarias were significantly (*p* < 0.05) promoted with an increase in MOLE levels with an optimum of 1.25 g MOLE/kg. It was noticed that final body weight, specific growth rate, and feed intake were increased with an increase in MOLE levels, and they are highly correlated. More so, dietary MOLE significantly evoked (*p* < 0.05) hematological profiles and antioxidant profiles of heteroclarias than the control group. Alanine aminotransferase was significantly reduced in fish fed MOLE-based diets compared to the fish fed a control diet, with their least value (27.27 IU/L) observed in fish fed a diet with 1.5 g MOLE/kg, while the highest was recorded in fish fed a control diet (32.03 IU/L). Also, non-specific immune profiles were greatly promoted by dietary supplementation. Additionally, fish survival post *A. hydrophila* infection was higher in fish fed a 2.0 g MOLE/kg diet (96.33%) than those fed a control diet (46.67%). This study revealed that MOLE administration enhanced the growth performance (linearly and quadratically), antioxidant (linearly), and innate immunity (linearly and quadratically) profiles of heteroclarias with an optimum level of 1.35 g/kg of diet. Likewise, its addition to the diets evoked the survival of fish against *A. hydrophila* infection.

## 1 Introduction

Aquaculture is a sustainable solution to the world’s problems of hunger, malnutrition, and declining health among the large human population (FAO, 2022), which has resulted in a progressive increase in the demand for fish (Adeshina et al., 2016). Hybrid catfish production has become common in various regions such as Africa, Asia, Europe, and the USA (Porto-Foresti et al., 2013). The hybrid African catfish (*Clarias gariepinus ♀ × Heterobranchus longifilis ♂*), also known as ‘heteroclarias’, has great market potential due to its ability to grow fast and attain higher weight, hardiness, high demand, and good taste. There are assertions that hybrid catfish are preferable to native catfish in terms of their improved aquaculture performances, including reduced feed conversion ratios, improved immunological responses, and increased metabolic enzyme activities (Porto-Foresti et al., 2013).

However, production of the hybrid catfish is hindered by the availability of quality feed and its high cost *Moringa oleifera* can be used as feed supplement crude protein, stimulate immune-stimulation, and confers antimicrobial properties in the fish diets (Tiamiyu et al., 2016).

Hybrid catfish production is greatly affected by *Aeromonas hydrophila* infection. This infection causes fin rot, hemorrhage, septicemia, furunculosis, and red sore disease and consequently leading to huge mortality of fish (Adeshina et al., 2019). It has been reported that *A. hydrophila* responsible for 60% mortality in catfish farming in Nigeria and Africa (Adeshina et al., 2024; Adeshina et al., 2018b), and therefore, protecting it from aeromonasis infection cannot be over emphasized for profitable venture. Also, fish farmers often use chemical additives and antibiotics in fish feed to prevent diseases and improve feed utilization to meet the ever-rising demands of fish production (Paray et al., 2024). The presence of antibiotic residue in aquaculture products has raised questions about food safety in addition to the hazard that these strains pose through the transmission of resistant genes to human infections (Rigos and Kogiannou, 2023). The increasing drive to prevent the use of chemotherapeutics in fish ushered in the need for more sustainable options for prevention and treatment (Easwaran et al., 2022). As a result of this, the identification of functional feed additives with organic origins has received increased attention recently.

One plant receiving a lot of attention as a possible phytogenic additive is *Moringa oleifera* (MO) due to its anti-inflammatory, hepatoprotective, antimicrobial, and antioxidant effects (Khalil et al., 2020; Tabassum et al., 2023; Bisht et al., 2020). The leaves of MO have a high crude protein levels and essential amino acids (Dongmeza et al., 2006) as well as many essential vitamins and minerals (Kou et al., 2018). Also, it contains some phytochemicals such as carotenoids, flavonoids, phenolics, alkaloids, and terpenoids (Ahmadifar et al., 2021). Despite the advantages of MO, the higher presence of antinutritional factors has limited its utilization. These antinutritional factors can be removed through proper processing such as extraction. However, hybrid catfish production is constrained by high feed costs and concerns over antibiotic use. MOLE is a promising natural feed additive with nutritional, antimicrobial, antioxidant, and immunostimulatory properties. Additionally, the extract from the leaves could help to increase the fish appetite, feed consumption and consequently higher growth. Furthermore, MOLE is expected to stimulate defense against pathogenic agents by boosting immune responses. However, there is limited information on the effects of *Moringa oleifera* leaf extract (MOLE) in hybrid catfish. The study examined the growth performance, physiological profiles, and protective potentials of MOLE against *Aeromonas hydrophila* in heteroclarias, *Clarias gariepinus ♀ × Heterobranchus longifilis ♂*.

## 2 Materials and methods

### 2.1 Plant collection, identification, and extract preparation

Fresh *Moringa oleifera* leaves (MOL) were obtained in a private garden in Tanke, Ilorin, Nigeria, and confirmed at the Herbarium Unit, Department of Plant Biology, University of Ilorin, Ilorin, Nigeria. The MOL was washed under running water and air-dried for 14 days under shade. The dried leaves were milled to fine powder form using a kitchen blender (Marchesoni 2L, Marcpro, China) and cold-extracted in ethyl acetate in a ratio of 1:10 (meal: solvent; weight/volume for 72 h (Ahmed et al., 2019; Adeniyi et al., 2021) to produce *Moringa oleifera* leaves (MOLE). The ethyl acetate was removed from the extract using a rotary evaporator (Smart H150-1000, Italy) and filtered through a Whatman paper no. 1. The MOLE was kept at -20 °C until required.

### 2.2 Phytochemical and phyto-constituents’ analyses

Phytochemical constituents of MOLE were quantified as follows: one gram of MOLE was added to 10 mL of petroleum ether and allowed for 15 min. The solution was filtered, and absorbance was read at a wavelength of 420 nm to measure terpenoids. Flavonoids were measured by adding one gram (1 g) of MOLE to 10 mL of 80% methanol. The solution was allowed to stand for 2 h and filtered into a pre-weighed petri dish. The solution was then dried in an oven at 40 °C. The petri dish was weighed to constant weight (Joshi et al., 2011). For tannin determination, 1 g of MOLE was mixed with 25 mL of a solvent mixture containing acetone and 10% glacial acetic acid in an 80:20 ratio. The mixture was allowed to stand for 5 h, after which the absorbance was measured at 500 nm using a spectrophotometer. A reagent blank was also prepared and analyzed. Tannic acid standards (10, 20, 30, 40, and 50 mg/100 g) were used to construct a calibration curve. The tannin concentration of the sample was then determined from the standard curve, taking into account the appropriate dilution factor. One (1) gram of sample (W) was weighed and added to 20 mL of 10% acetic acid in ethanol. The solution was shaken and allowed to stand for 4 h and filtered. The solution was evaporated to a quarter of its original volume, and one drop of concentrated ammonia was added. The precipitation was then filtered through a filter paper (W1) and dried in an oven at 60 °C. The weight of the filter paper was taken after constant weight (W2) (Joshi et al., 2011) to determine alkaloids. $\text{Alkaloids}\left(\%\right)=\frac{\left({\text{W}}_{2}-{\text{W}}_{1}\right)}{\text{W}}\times 100$. Saponins were measured by adding one (1) gram of sample into 5 mL of 20% ethanol and putting it in a water bath at 55 °C for 4 h. The solution was filtered through a filter paper (Whatman No. 1), and the residue was washed with 20% ethanol twice. The solution was concentrated to 5 mL in the oven. Five (5) mL of petroleum ether was added to the concentrated extract inside a separating funnel. The petroleum ether layer was discarded, and 3 mL of butanol was added to it, then washed with 5 mL of 5% sodium chloride. Butanol was later poured into a weighed Petri dish and evaporated to dryness in an oven at 60 °C and weighed, while steroids were measured by adding 100 mL of ethanol to five grams of the sample. Exactly 0.1 mL ammonium hydroxide was added to the solution to take the pH to 9.1. Two (2) mL petroleum ether, 3 mL acetic anhydride and concentrated H_2_SO_4_ were added to the solution, and values were read from the absorbance at 420 nm (Joshi et al., 2011). Bioactive compounds, on the other hand, were evaluated using gas chromatography (GC, Agilent 5975, Avondale, PA, USA)/mass spectrometry (MS, Agilent 7890A, Avondale, PA, USA) containing a 5-metre-long HP column (0.25 cm internal diameter) (Adeshina et al., 2018a).

The metabolites and phytoconstituents were shown in **Tables 1**, **2**, respectively.

**Table 1:** Quantitative results of secondary metabolites in the MOLE.

Parameters	(mg/100g)
Alkaloids	175.00 ± 15.00
Terpenoids	88.33 ± 2.89
Tannins	221.67 ± 7.64
Steroids	156.67 ± 10.41
Sapoinins	173.08 ± 10.41
Flavonoids	58.33 ± 10.41

Values are presented as mean ± Standard error.

**Table 2:** Ten (10) major phyto-constituents identified in MOLE.

Name of compound	RT ^1^ (min)	MF ^2^	MW (g mol-^1^>) ^3^	Conc. (%) ^4^	QP (%) ^5^
Furaneol	6.672	C_6_H_8_O_3_	128	25.05	94
2-Oxepanone	6.226	C_6_H_10_O_2_	114	10.10	99
Benzeneacetaldehyde	6.587	C_8_H_8_O	120	9.15	90
Thiophene	9.145	C_4_H_6_S	86	5.24	91
Maltol	8.541	C_6_H_8_O_4_	144	25.98	95
Furopyranone	5.623	C_6_H_8_O_4_	144	3.23	90
dl-mevalonicacidlactone	10.125	C_6_H_10_O_3_	130	1.23	89
Docosylethylether	21.091	C_24_H_50_O	354	1.56	90
Butanoicacid,3,3Dimethyl-	10.975	C_6_H_12_O_2_	116	1.03	91
2(3H)-Furanone,5 acetyldihydro	8.934	C_6_H_8_O_3_	128	2.12	92

^1^ RT, retention time; ^2^ MF, molecular formula; ^3^ MW, molecular weight, ^4^ Conc. (%): percentage concentration; ^5^ Qual. Peak, Quality Peak.

### 2.3 Diets preparation

Five isonitrogenous diets (400 g/kg crude protein) were prepared and supplemented with MOLE at 0.0, 0.5, 1.0, 1.5, or 2.0 g/kg (**Table 3**). Briefly, all ingredients were then mixed with distilled water (100 mL/kg), pelleted into 2.0 mm size using a pelletiser (Model HSDGP-60, Zhengzhou E.P. Machinery, Zhengzhou, China), and air-dried at a room temperature. The dried diets were kept at -20 °C until use. Furthermore, the diets were reconstituted at every fortnight interval to ensure nutrient retention and prevent extract deterioration.

**Table 3:** Ingredients and proximate composition (g/kg diet on dry matter basis) of diets containing different levels of *M. oleifera* leaves extract (MOLE).

Ingredients	MOLE levels (g/kg)				
	0.0	0.5	1.0	1.5	2.0
Fishmeal (Crude protein 65%)	200.0	200.0	200.0	200.0	200.0
Soybean meal (Crude protein 44%)	300.0	300.0	300.0	300.0	300.0
Groundnut cake (Crude protein 45%)	300.0	300.0	300.0	300.0	300.0
Maize (Crude protein 9%)	110.0	110.0	110.0	110.0	110.0
Soybean oil	30.0	30.0	30.0	30.0	30.0
Starch	40.0	39.5	39.0	38.5	38.0
Premix 1	20.0	20.0	20.0	20.0	20.0
MOLE	0.0	0.5	1.0	1.5	2.0
Total	1000.0	1000.0	1000.0	1000.0	1000.0
Proximate composition (g/kg)					
Dry matter	932.0	932.0	932.0	932.0	933.0
Crude protein	406.0	405.0	408.0	408.0	408.0
Ether extract	104.0	103.0	103.0	102.0	102.0
Total ash	77.0	76.0	78.0	78.0	78.0
Crude fiber	109.0	105.0	110.0	111.0	111.0

^1^ Premixes = HI-MIXAQUA (Fish) each 1 kg contains as follows. Vitamin A: 4,000,000 International Unit (IU); vitamin D3: 8,00,000 IU; vitamin E: 40,000 IU; vitamin K3: 1,600 mg; vitamin B1: 4,000 mg; vitamin B2: 3,000 mg; vitamin B6: 3,800 mg; vitamin B12: 3 mcg; nicotinic acid: 18,000 mg; pantothenic acid: 8,000 mg; folic acid: 800 mg; biotin: 100 mcg; choline chloride: 120,000 mg; iron: 8,000 mg; copper: 800 mg; manganese: 6,000 mg; zinc: 20,000 mg; iodine: 400 mg; selenium: 40 mg; vitamin C (coated): 60,000 mg; inositol: 10,000 mg; cobalt: 150 mg; lysine: 10,000 mg; methionine: 10,000 mg; antioxidant: 25,000 mg.

### 2.4 Fish culture

Heteroclarias, *Clarias gariepinus ♀ × Heterobranchus longifilis ♂* (n = 350; mean weight 9.3 ± 0.2 g), corresponding to fingerlings, were acquired and acclimated for 14 days during which fish were fed a control diet. Then, 300 fish were randomly distributed into 15 aquaria/experimental units (100 L capacity, each treatment has three replicates), where each aquarium contained 20 fish connected to the well-aerated water from the overhead tank. The fish were fed experimental diets for 8 weeks two times (7:00 and 17:00 h) a day up to satiation. The tank’s water was monitored daily using a digital water quality parameter (Photoic 20; Labtech International) and a mercury-in-glass thermometer. The dissolved oxygen (DO) values were between 5.7 and 5.8 mg/L (DO), and pH was between 7.5 and 7.8, while temperature ranged from 27.8 to 29.2 °C. These values were within the recommended values for raising catfish (Boyd and Tucker, 2012).

### 2.5 Zoo-techniques measurement

At the end of the feeding trial, fish from each tank were weighed with the aid of a sensitive scale (Mettler Toledo FB602, Jenway UK) and growth and feed utilization were calculated as follows:

Body weight gain (BWG, g) = final body weight (FBW, g)–initial body weight (IBW, g);

Daily body weight gain (DBW, g/day) = BWG (g)/Experimental period (T);

Specific growth rate (SGR; %/day) = 100 [LN FBW (g) − LN IBW (g)]/T;

Feed intake (FI) = the summation of the offered feed (g) to fish throughout the experiment;

Feed conversion ratio (FCR) = FI (g)/BWG (g);

Fish survival rates (SR) (%) = 100 (final number of fish/initial number of fish).

### 2.6 Evaluation of short-chain fatty acids, digesta pH and viscosity

The digesta pH was measured *in situ* by inserting the probe of the pH meter (Photoic 20, Labtech International Ltd, Heathfield, UK) into the intestinal digesta of each fish, and the readings were recorded. Viscosity was measured by centrifuging the feces at 28 °C and 3000 x *g* for 10 min (Model: LC400, Joanlab Laboratories, China). Then, the supernatant was placed in a viscometer (NDJ-9S, RaeSung Inc., India) using a 50.0 per sec shear rate to assess viscosity (centipoise, cP) (Leenhouwers et al., 2007). The short-chain fatty acids (SCFAs) (acetic, propionic, and butyric acids) were examined using gas chromatography (Agilent 7890A) (Avondale, PA, USA) equipped with an HP column of 5 m long (Agilent 190915-433HP-5M) operated at an ionization energy of 70 eV and a flame ionization detector, after acidification with 1 M o-phosphoric acid p.a. (Ref. 100,573, Merck^©^) and fortification with a mixture of free volatile acids (Ref. 46,975, Supelco^©^). In order to separate the analytes in a chromatographic run of 11.5 minutes, an aliquot of 1 μL of each sample was injected (split ratio of 40:1) using helium as a carrier gas with a linear velocity of 42 cm/s. The initial column temperature was 40 °C, and the injector and detector temperatures were 250 °C and 300 °C, respectively. Starting at 40 °C min^-^¹, the column temperature ramp progressed from 40 to 120 °C, then from 120 to 180 °C at 10 °C min^-^¹, and finally from 180 to 240 °C at 120 °C min^-^¹, maintaining the temperature at 240 °C for an additional 3 minutes. The aforementioned conditions were used to analyze calibrated dilutions of the WSFA-2 standard (Ref. 47,056, Supelco^®^) and glacial acetic acid (Ref. 33,209, Sigma-Aldrich^®^) in order to quantify the analytes. The software GCsolution v. 2.42.00 (Shimadzu, Kyoto, Japan) was used to identify and integrate the peaks.

### 2.7 Evaluation of haemato-biochemical and liver enzyme profiles of the fish

Two fish from each tank were anesthetized using tricaine methanesulfonate buffered with sodium bicarbonate (MS222, Sigma-Aldrich, USA; 30 mg/L). Then, a needle and syringe were used to puncture the caudal veins to draw blood. The blood was then split in half. The first half was drawn using the Brown, 1980 method and dispensed into the lithium-heparinized bottles to determine the number of red blood cells (RBC), white blood cells (WBC), and platelets. Also, the blood samples were mixed by inverting for 20 times. The capillary tubes were filled with the blood samples and centrifuged at 3000 rpm for 5 minutes in a Kawskley micro-hematocrit centrifuge, England. The actual PCV values were obtained using hematocrit reader and expressed in percentage (Boyd and Tucker, 2012). Hemoglobin was measured in accordance with Vankampen and Ziglstra (1961), and Wright-Giemsa stain was used to perform differential counts (lymphocytes, heterocytes, monocytes, eosinophils, and basophils). Mean Cell Hemogblobin (MCH) was determined as the average of Hb in pictograms of a simple RBC was determined using $\text{MCH}\left(\text{pg}\right)=\frac{\text{Hb}\times 10}{\text{RBC}}$, mean cell hemogblobin concentration (MCHC) was estimated as percentage of Hb of packed RBC was determined using $\text{MCHC}\left(\text{g}/\text{dL}\right)=\frac{\text{Hb}}{\text{PCV}}\times 100$, while mean cell volume (MCV) as average volume of a single cell was determined using $\text{MCV}\left(\text{Fl}\right)=\frac{\text{PCV}\times 10}{\text{RBC}}$. On the other hand, the serum was extracted by centrifuging (3000 x g) the’ clotted second portion of the blood for 10 minutes at 35 °C. Then, using the colorimetric method (Reitman and Frankel, 1957), the activities of alkaline phosphatase (ALP), aspartate aminotransferase (AST), and alanine aminotransferase (ALT) were assessed with the use of Randox commercial kits (Randox^®^ Laboratories Ltd. Crumlin, County Antrim, United Kingdom).

### 2.8 Determination of anti-oxidant and immune parameters

For determination of glutathione peroxidase (GPx), superoxide dismutase (SOD) catalase (CAT), and malondialdehyde (MDA) the liver tissues were extracted and cleaned with distilled water. A Potter- Elvehjem glass/Teflon homogenizer was then used to homogenize 1.0 g of the tissue in 9 mL of cold phosphate buffer saline (0.1 M pH 7.4). To quantify the antioxidant parameters, diagnostic reagent sets from Randox^®^ Laboratories in Crumlin, County Antrim, UK, were utilized for glutathione peroxidase (GPx) (CAT. No.: RS504; Detection limit: Spectrophotometer (412 nm)) while for superoxide dismutase (SOD, U mg protein^-^¹; Cat. No. EIASODC), catalase (CAT, U mg protein^-^¹; Cat. No. EIACATC), and malondialdehyde (MDA, nmol mg protein^-^¹; Cat. No. EEA015) were determined using Invitrogen™ ThermoFisher Scientific Inc. diagnostic kits. To assess the activities of glutathione-S-transferase (GST), 1 mM glutathione was conjugated with 1 mM 1-chloro-2,4-dinitrobenzene (CDNB) at 340 nm. Moreover, the activities of serum lysozyme and respiratory burst were evaluated using the colorimetric method and the nitroblue tetrazolium dye method (Secombes, 1990; Brown, 1980). Briefly, lysozyme activity was calculated from a standard curve prepared with lysozyme from chicken egg white. Serum lysozyme activity was determined using a colorimetric assay following the method described by Grinde (1989). Briefly, serum samples were incubated with a suspension of *Micrococcus lysodeikticus* cells as the substrate. The reduction in turbidity resulting from bacterial cell wall lysis was measured spectrophotometrically at 600 nm using a microplate reader (HINOTEK, AMR-100). A standard curve was prepared using lysozyme from chicken egg white (Sigma-Aldrich, Cat. L7651), and lysozyme activity was calculated from the standard curve and expressed as


$$Lysozyme\;concentration\left(\frac{\mu g}{mL}\right)=\frac{Activity\left(\frac{U}{mg}\right)\times serum\;protein\left(\frac{mg}{mL}\right)}{Specific\;activity\;\frac{U}{\mu g}}.$$

Where the chicken egg-white lysozyme specific activity of approximately 40,000 U/mg lysozyme (40 U/µg) and total serum protein = 30 mg/mL (Grinde, 1989).

Respiratory burst activity (RBA) was measured using diagnostic reagent kits (Randox, London, UK). RBA was assessed using the nitroblue tetrazolium (NBT) reduction assay. The assay is based on the reduction of yellow NBT to blue formazan by intracellular reactive oxygen species generated by activated phagocytic cells. Briefly, 100 µL of whole-blood suspension was dispensed into each well of a 96-well microtiter plate (MaxiSorp™ 96-, Nalge-Nunc, Hereford, UK) and incubated with NBT solution (concentration 1 mg/mL) at 37 °C for 15 min. After incubation, the cells were fixed and the formazan precipitate was solubilized using 5% dimethyl sulfoxide. Absorbance was measured at 570 nm using a microplate reader (MaxiSorp™ 96-, Nalge-Nunc, Hereford, UK). Respiratory burst activity was expressed as optical density (OD) values or calculated according to the manufacturer’s instructions as follows:


$$\text{RBA}={\text{OD}}_{\text{sample}}-{\text{OD}}_{\text{blank}}$$

All measurements were performed in triplicate, and the mean value was used for statistical analysis.

### 2.9 Bacterial challenge

Following eight weeks of feeding trials, ten fish from each tank underwent a 24-h starvation period prior to the challenge test. A molecularly characterized isolate of *Aeromonas hydrophila* (ATCC 4356) was incubated for 24 h at 35 °C, centrifuged, and then re-suspended at 5.0 × 10^5^ CFU/mL in peptone water (0.1%). The bacterial culture of *Aeromonas hydrophila* (ATCC 4356) was grown in nutrient broth for 24 h at 35 °C. Following incubation, the culture was centrifuged at 3,000 × g for 10 min, and the resulting pellet was washed and re-suspended in sterile 0.1% peptone water. The bacterial concentration was initially adjusted spectrophotometrically to approximately 1.0 × 10^8^ CFU/mL using the turbidity equivalent to a 0.5 McFarland standard. Subsequently, serial ten-fold dilutions were prepared in sterile 0.1% peptone water to obtain the desired concentration of 5.0 × 10^5^ CFU/mL. The final bacterial density was confirmed by the standard plate count method on nutrient agar and expressed as colony-forming units per milliliter (CFU/mL). Before the challenge trial, the pathogenicity of the *A. hydrophila* isolate (ATCC 4356) was verified in healthy fish. A group of fish was intraperitoneally injected with the bacterial suspension, while a control group received sterile 0.1% peptone water. The fish were monitored daily for clinical signs, behavioral abnormalities, and mortality for 14 days. Re-isolation and identification of *A. hydrophila* from the kidney and liver of moribund fish were conducted to confirm the cause of infection. The isolate was considered pathogenic when it consistently induced characteristic clinical signs and mortality in the challenged fish, while no mortality or disease symptoms were observed in the control group. After injecting *A. hydrophila* (0.1 mL; 5.0 × 10^5^ CFU/mL) intraperitoneally (IP), the fish were fed the appropriate food and observed for a period of two weeks.

### 2.10 Statistical analysis

The homogeneity of variances was assessed using Bartlett’s test, and the normality of the data’s distribution was verified using Kolmogorov-Smirnov’s test. Each aquarium/tank was treated as an experimental unit and used for statistical analysis. After that, a one-way analysis of variance was used to examine the collected data. Following Duncan’s multiple range test at a 5% probability level, significant differences in means were subjected to orthogonal linear and quadratic comparison. The optimal level of dietary MOLE was then determined using polynomial regression analysis while survival data were analyzed using the Kaplan–Meier estimator in SPSS version XX. Differences among treatment groups were assessed using the log-rank (Mantel–Cox) test. Statistical significance was set at *p* < 0.05 with the use of a statistical package (SPSS program V20) (Dytham, 2011).

## 3 Results

### 3.1 Performance of growth and feed utilization

**Figure 1:**
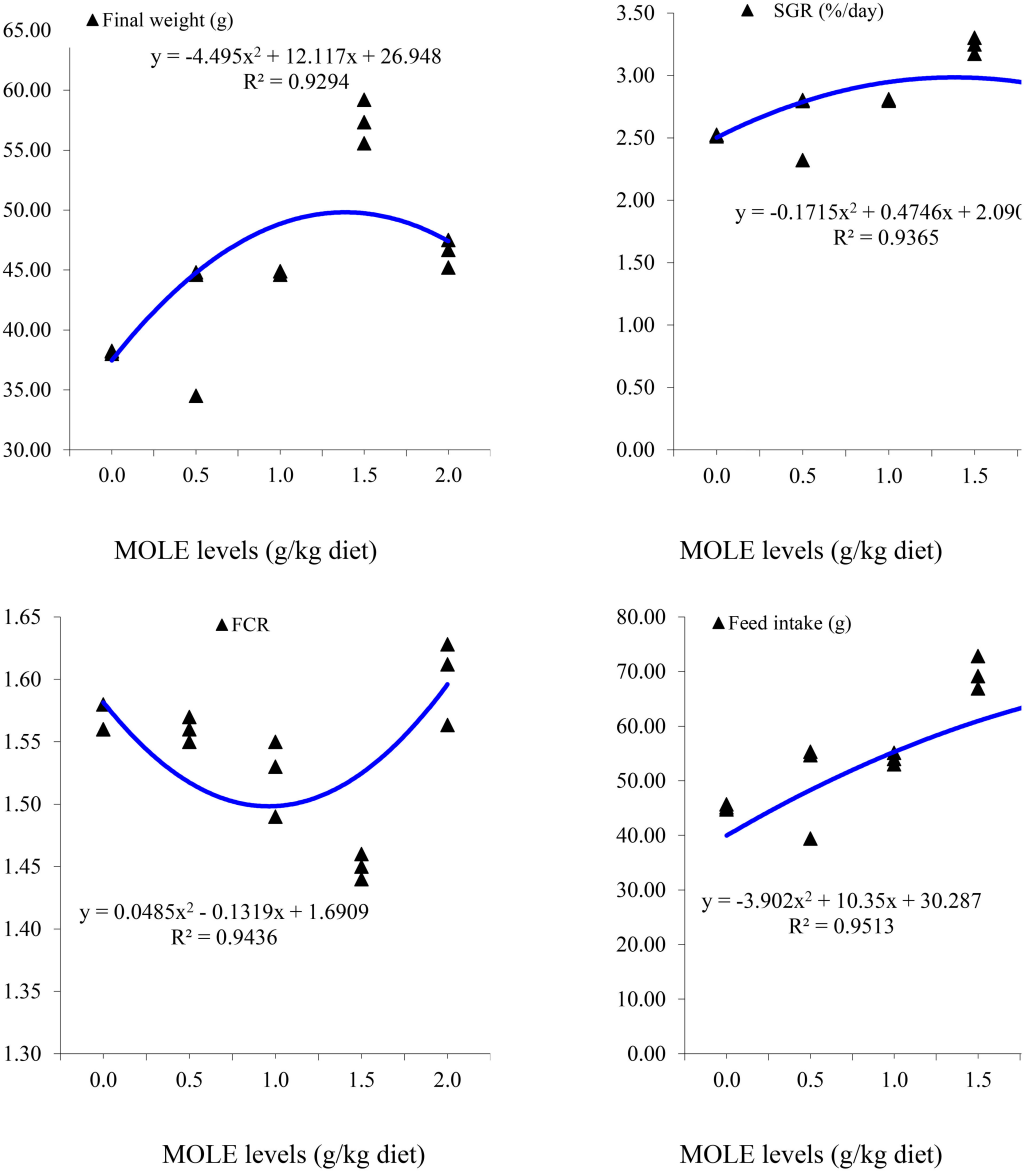
The relationship between FW (g), SGR (%/day), FCR, and FI (g) of heteroclarias, juveniles fed MOLE levels for 56 days (n = 3; *p<* 0.05; second order polynomial regression). FW, Final weight; SGR, Specific growth rate; FI, Feed intake; MOLE, *Moringa oleifera* leave extract.

Growth performance of Heteroclarias was significantly (*p* < 0.05) promoted in fish fed a MOLE-based diet than the control. Linearly and quadratically, dietary MOLE greatly improved FBW, BWG, DBW, SGR, and FI compared to the control group (*p* < 0.05; **Table 4**) in a dose-dependent manner. Their maximum values were observed in the fish fed a 2.0 MOLE g/kg diet. The feed conversion ratio (FCR), on the other hand, significantly (*p* < 0.05) reduced due to the addition of MOLE in the diet of heteroclarias. FSR was not significantly altered (*p* > 0.05; **Table 4**) when fed dietary MOLE. The relationships between FBW, SGR, FCR, and FI, and MOLE levels (**Figure 1**) were best expressed by the second-order polynomial regression as follows: $y=-4.495{\text{x}}^{2}+12.117x+26.948\left(R^{2}=0.9294\right)$, $y=-0.1715{\text{x}}^{2}+0.4746x+2.0906\left(R^{2}=0.9365\right)$, $y=0.0485{\text{x}}^{2}-0.1319x+1.6909\;\left(R^{2}\;=0.9436\right)$, and $y=-3.902{\text{x}}^{2}+10.35x+30.287\left(R^{2}=0.9513\right)$. These relationships evoked that the optimum MOLE level for heteroclarias is found to be 1.35 g/kg diet.

**Table 4:** Growth performance and feed utilization of heteroclarias fed with diets enriched with different levels of *Moringa oleifera* leaves extract (MOLE) for 56 days.

Parameters ^1^	MOLE levels (g/kg)					SEM ^2^	p-values	
	0.0	0.5	1.0	1.5	2.0		Linear	Quadratic
IBW (g)	9.30 a	9.33 a	9.30 a	9.30 a	9.33 a	0.009	0.580	0.671
FBW (g)	38.17 c	41.30 bc	44.80 b	57.37 a	46.46 b	2.252	<0.001	<0.001
BWG (g)	28.87 c	31.97 bc	35.50 b	48.07 a	37.13 b	2.254	<0.001	<0.001
DBW (g/day)	0.52 b	0.57 b	0.63 b	0.86 a	0.66 b	0.045	<0.001	<0.001
SGR (%/day)	2.50 c	2.63 bc	2.80 b	3.23 a	2.87 b	0.086	<0.001	0.001
FI (g)	45.20 d	49.80 cd	54.03 c	69.63 a	59.43 b	3.715	<0.001	<0.001
FCR	1.57 a	1.56 a	1.52 b	1.45 b	1.60 a	0.011	0.004	0.001
FSR (%)	93.33 a	93.33 a	98.33 a	100.00 a	98.33 a	1.259	0.319	0.503

Means having the same letter in the same row are not significantly different at *P* > 0.05.

^1^ IBW, Initial body weight (g); FBW, Final body weight (g); BWG, Body weight gain (g); DBW, Daily body weight gain (g/day), SGR, Specific growth rate (SGR; %/day), FI, Feed intake (g), FCR, Feed conversion ratio; FSR, Fish survival rates (%).

^2^ SEM, Standard error mean.

### 3.2 Haematological profiles

The study showed haematological profiles of Heteroclarias fed MOLE-based diets were linearly and quadratically significantly altered (*p* < 0.05; **Table 5**). The PCV, Hb, RBC, WBC, and PLT were both linearly and quadratically different among the fish fed experimental diets. Their highest values were recorded in fish diets enriched with 2.0 g MOLE/kg diet, while the lowest corresponding values were obtained in fish fed a control diet. The variations in the haematological profiles were in a dose-dependent manner (**Table 5**). However, dietary MOLE did not significantly alter the differential cell count of LYM, HET, MON, EOS, and EOS (*p* > 0.05; **Table 5**). Also, MCH and MCV were significantly altered (*p* < 0.05; **Table 5**) in fish fed with fortified diets while MCHC was not significantly differed (*p* > 0.05; **Table 5**).

**Table 5:** Hematological profiles of heterolalias fed with diets enriched with different levels of *Moringa oleifera* leaves extract (MOLE) for 56 days.

Parameters ^1^	MOLE levels (g/kg)					SEM ^2^	p-values	
	0.0	0.5	1.0	1.5	2.0		Linear	Quadratic
PCV (%)	16.70 c	21.33 b	21.43 b	22.63 ab	22.83 a	0.616	<0.001	0.001
Hb (g/dL)	4.63 c	6.37 b	6.50 ab	6.90 a	6.90 a	0.233	<0.001	<0.001
RBC (x106/μL)	1.23 c	1.53 b	1.53 b	1.83 a	1.93 a	0.068	<0.001	<0.001
WBC (x103/μL)	149.23 b	191.30 a	186.83 a	187.63 a	189.00 a	0.068	<0.001	<0.001
PLT (x106/μL)	166.03 d	200.30 d	221.00 b	269.03 a	273.93 a	11.053	<0.001	0.001
LYM (%)	51.67 b	57.33 ab	58.67 a	59.33 a	61.00 a	1.125	0.050	0.336
HET (%)	42.57 a	34.67 a	37.00 a	35.00 a	34.33 a	1.167	0.069	0.327
MON (%)	3.00 a	3.00 a	3.00 a	1.67 a	1.67 a	0.364	0.165	0.682
EOS (%)	2.67 a	3.00 a	3.17 a	3.33 a	3.00 a	0.316	0.674	0.556
BAS (%)	0.67 a	0.67 a	1.00 a	0.67 a	0.00 a	0.163	0.275	0.174
MCH (pg)	37.64 b	41.63 a	42.48 a	37.70 b	35.75 b	2.775	0.007	0.151
MCHC (g/dL)	27.73 a	29.86 a	30.33 a	30.49 a	30.22 a	1.882	0.221	0.145
MCV (FI)	135.77 b	139.41 a	140.07 a	123.66 c	118.29 d	8.677	0.002	0.241

Means having the same letter in the same row are not significantly different at *P* > 0.05.

^1^ PCV, Packed cell volumes (%); Hb, Hemoglobin (g/dL); RBC, Red blood cell (x10^6^/μL); WBC, White blood cell (x10^3^/μL); PLT, Platelets (x 10^3^/μL); LYM, Lymphocytes (%); HET, Heterocytes (%), MON, Monocytes (%); EOS, Eosinophils (%), BAS, Basophils (%).

^2^ SEM, Standard error mean.

### 3.3 Antioxidant activity and liver enzymes

**Figure 2:**
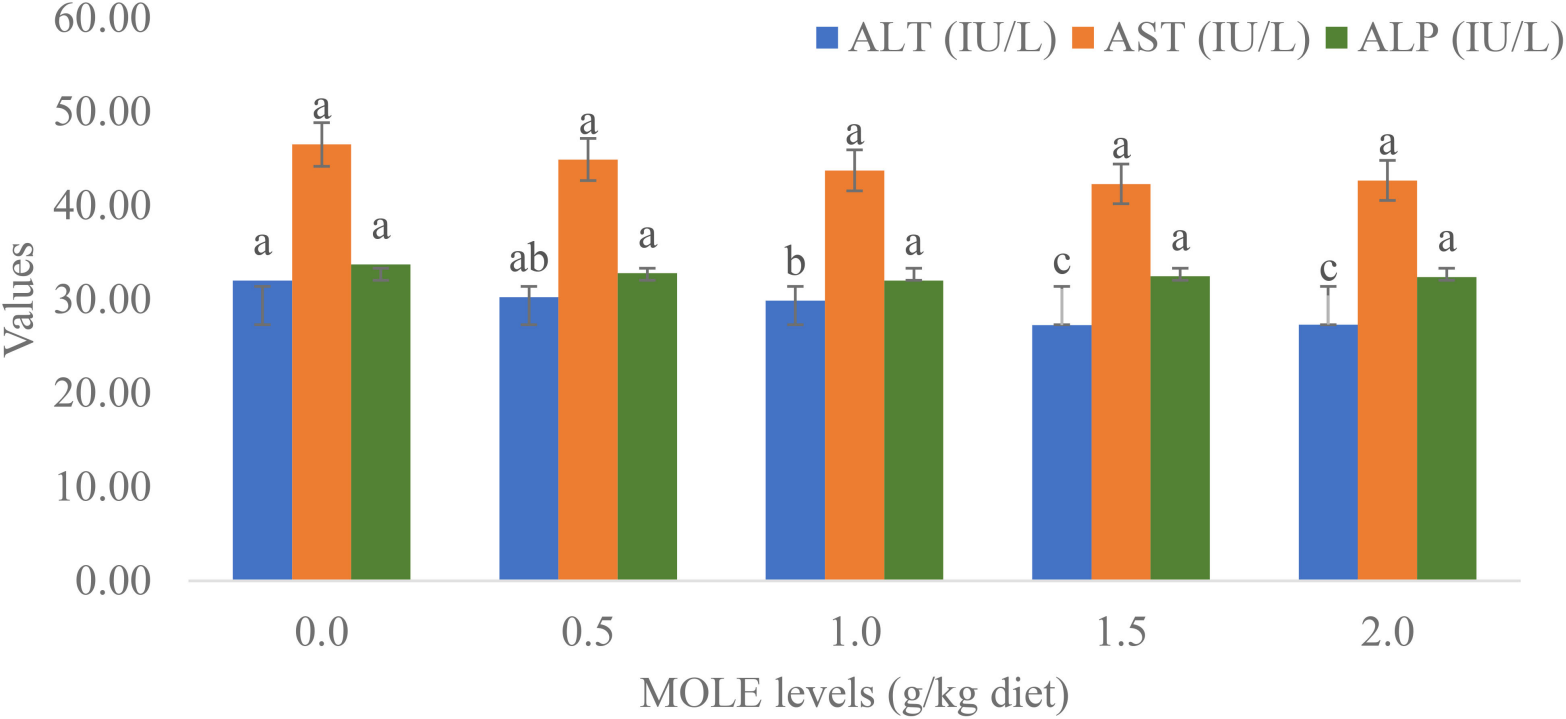
Liver enzymes activity of heteroclarias fed with diets enriched with different levels of *Moringa oleifera* leaves extract (MOLE) for 56 days (n = 3; ANOVA; Duncan multiple range test). MOLE = *Moringa oleifera* leave extract Bars of the same patterns/color with different superscript are significantly different (*p* < 0.05).

Antioxidant profiles of heteroclarias fed MOLE-based diets showed linear significant (*p* < 0.05; **Table 6**) improvements but not quadratic (*p* > 0.05; **Table 6**). The improvements in the levels of GPx, SOD, CAT, and GST were directly proportional to the MOLE inclusion levels in the diet, showing their highest in fish-fed diets containing 2.0 g MOLE/kg diet. On the other hand, reduction in the levels of MDA (*p* < 0.05) was caused by dietary MOLE. Fish fed 2.0 g MOLE/kg has the lowest MDA level, while the highest was observed in fish fed a control diet (**Table 6**). Regarding the liver enzyme activity of heteroclarias fed graded MOLE for 56 days, ALT level was significantly reduced with an increase in dietary MOLE levels, and their maximum values were detected in fish fed a control diet, with their corresponding lowest values obtained in fish fed a 2.0 MOLE g/kg diet (*p* < 0.05; **Figure 2**). However, AST and ALP levels were not linearly and quadratically significantly affected due to dietary MOLE (*p* > 0.05).

**Table 6:** Antioxidant profiles of heteroclarias fed with diets enriched with different levels of *Moringa oleifera* leaves extract (MOLE) for 56 days.

MOLE levels (g/kg)		Parameter ^1^							
		GPx (U mg protein^-^¹)	MDA (U mg protein^-^¹)		SOD (U mg protein^-^¹)		CAT (U mg protein^-^¹)		GST, U mg protein^-^¹)
0.0		51.20 b		5.40 a		20.40 c		23.13 c	30.43 b
0.5		55.33 a		4.13 b		24.40 b		25.90 b	36.27 a
1.0		56.83 a		3.60 c		27.13 a		26.93 a	36.83 a
1.5		62.43		2.80		31.27		34.60	43.47
2.0		62.73		2.73		34.17		35.10	44.37
SEM 2		1.203		0.289		1.340		1.317	1.394
p- values	Linear	<0.001	<0.001		0.001		<0.001		0.001
	Quadratic	0.206	0.104		0.892		0.432		0.153

Means having the same letter in the same column are not significantly different at *P* > 0.05.

^1^ GPx, Glutathione peroxidase. (IU/L); MDA, Malondialdehyde (µmol/mg protein), SOD, Superoxide dismutase (IU/L), CAT, Catalase (IU/L), GST, Glutathione-S-transferase (nmol/L).

^2^ SEM, Standard error mean

### 3.4 Immunity and survival after infection

**Figure 3:**
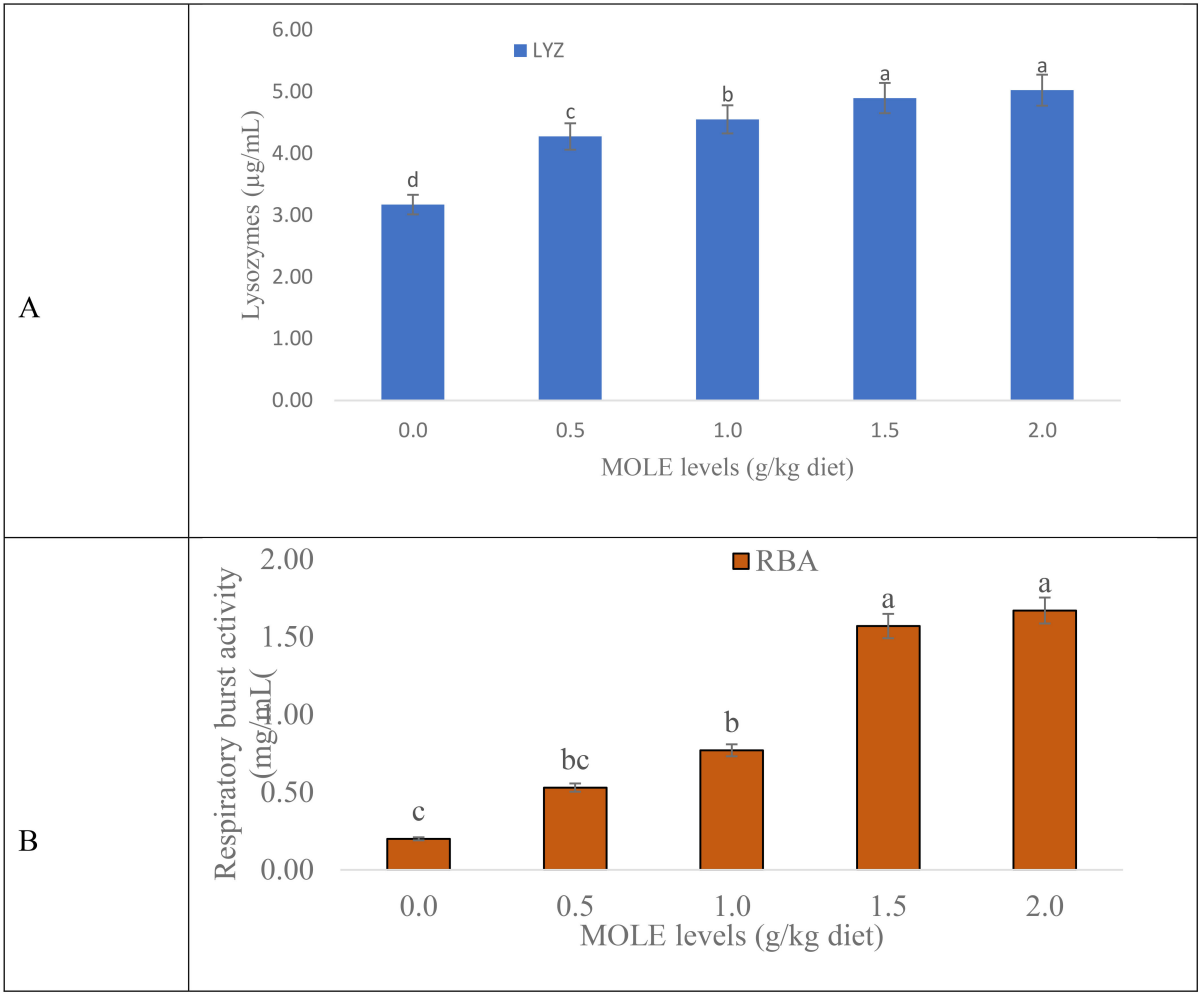
Lysozymes, and respiratory burst activity of heteroclarias fed with diets enriched with different levels of *Moringa oleifera* leaves extract (MOLE) for 56 days. MOLE = *Moringa oleifera* leave extract (n = 3; ANOVA; Duncan multiple range test). LYZ, Lysozymes; RBA, Respiratory burst activity Bars of the same patterns/color with different superscript are significantly different (*p* < 0.05).

**Figure 4:**
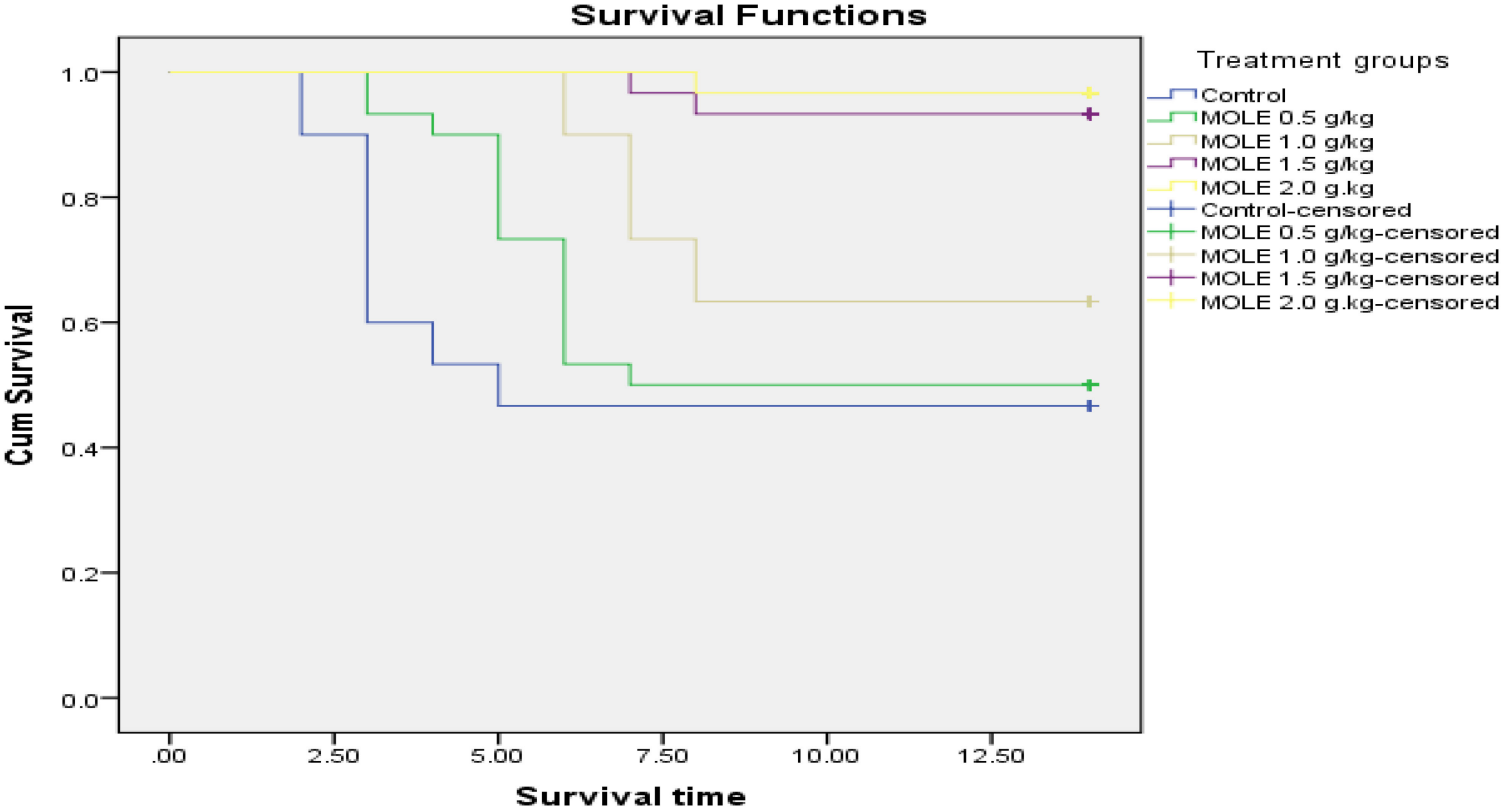
Kaplan-Meier survival curve survival of heteroclarias fed with diets enriched with different levels of *Moringa oleifera* leaves extract (MOLE) for 56 days and exposed to *Aeromonas hydrophila* infection for 14 days.-.

Non-specific immunity of heteroclarias was presented in **Figure 3**. There were linearly and quadratically significant differences in the LYZ and RBA levels of the fish (*p* < 0.05). The highest LYZ and RBA were obtained in fish fed with a 2.0 g MOLE/kg diet, while the lowest values were detected in fish fed on the control diet (**Figures 3a, b**). The survival rate of fish during the 14-day post-A. *hydrophila* infection was shown in **Table 7**; **Figure 4**. The inclusion of MOLE in the diets for Heteroclarias evoked survival post-bacteria challenge in an inclusion-dependent order. **Table 7**; **Figure 4** revealed fish fed enriched diets survived better than their control counterpart. At day 8 post-infection, 93.3% fish fed with diet fortified with 1.5 MOLE g/kg survived while only 46.5% survival was recorded in the control group at day 5 (*p<* 0.05). Kaplan–Meier survival analysis revealed significantly higher survival probabilities in fish fed the MOLE-supplemented diet compared with the control group (Log-rank test, χ² = 38.402, *p* = 0.001). The survival curve showed a reduced cumulative mortality throughout the challenge period in the supplemented groups.

**Table 7:** Survival prbaility of heteroclarias fed with diets enriched with different levels of *Moringa oleifera* leaves extract (MOLE) for 56 days and exposed to *Aeromonas hydrophila* infection for 14 days.

Time (days)	MOLE levels (g/kg)					P value
	0.0	0.5	1.0	1.5	2.0	
2	0.900					
3	0.600	0.933				
4	0.533	0.900				
5	0.467	0.733				
6		0.533	0.900			
7		0.500	0.733	0.967		
8				0.933	0.967	
11			0.633			<0.001

## 4 Discussion

A significant increase in final body weight and specific growth rate was observed in Heteroclarias fish when fed with MOLE-enriched diets, specifically at 1.0 g/kg and 2.0 g/kg levels, with 1.35 g MOLE/kg diet as the optimum inclusion level. In this study, fish fed MOLE-based diets expressed great growth performance when compared to the control group. This result indicated that MOLE induced appetite in Heteroclarias unscored more feed consumption and, consequently, better growth. Significantly enhanced growth and FI in Nile tilapia were reported when fed dietary MOLE at inclusion levels ranging from 0.5 to 2.0 g/kg of diet with an optimum dose of 0.5 g MOLE/kg (Kamble et al., 2024). The difference in optimum level (1.35 g MOLE/kg diet) noticed in this study versus 0.5 g MOLE/kg diet recorded in Nile tilapia (Kamble et al., 2024) may be attributed to the different species used for studies because tilapia has the ability to utilize a plant-based diet more than catfish; thus, a lower optimum dosage level is not unexpected. The lower FCR, especially in fish fed a 1.5 g MOLE/kg diet, revealed that nutrients in the diet were well absorbed and utilized in this group. FCR status depicted that the growth-promoting activity of MOLE is directly linked with the bioavailability of its nutrients instead of feed digestibility. Furthermore, furaneol is a natural aromatic compound that enhances feed palatability, while maltol is a flavor enhancer known to stimulate feed intake (Conti et al., 2023). Benzene acetaldehyde is another volatile compound contributing to aroma and taste, which can improve feed attraction, consumption, and palatability. Therefore, the higher FI, lower FCR, and finally enhanced growth performance recorded in fish fed MOLE based diets can be associated with the presence of active compounds like furaneol, maltol, and benzeneacetaldehyde. Plant based extracts enhanced the growth rates in silver barb *Barbonymus gonionotus* (Farhad et al., 2023) and *Clarias gariepinus* (Paray et al., 2024) while maintaining steady and relatively lower FCR values. Interestingly, survival rates of heteroclarias faced a slight decline at 2.0 g MOLE/kg compared to when fed a diet containing 1.5 g MOLE/kg. It indicates that MOLE enhances growth rates at higher doses but may induce detrimental effects beyond a threshold limit. These results intensify the use of the optimum level of plant extracts in feed formulation for the proper growth and survival of cultured fish.

Hematological evaluation is a good indicator of evaluation of the nutritional status and health status of fish. As shown in this study, dietary supplementation with MOLE significantly influenced the hematological parameters of Heteroclarias, demonstrating its potency as a functional feed additive, occurring in a dose-dependent manner. Hematological profiles including PCV, Hb levels, and RBC counts were significantly increased, highlighting the positive influence of MOLE on fish health by improving blood profiles. MOLE also enhanced the oxygen-carrying capacity and overall vitality of heteroclarias. Addition of MOLE in the diets of Nile tilapia (Kamble et al., 2024) significantly boosted the activity of hematological parameters. The observed increase in PCV, Hb, RBC, WBC, and PLT in fish fed on MOLE-based diets, particularly at a 2.0 g/kg diet, advocates improved hematopoiesis and overall health status through enhancing oxygen transport capacity and immunity. These improvements may be connected to the presence of bioactive compounds such as flavonoids in the MOLE. Studies have shown that secondary metabolites such as flavonoids’ presence in phytobiotics promote erythropoiesis in fish, resulting in stress resistance and recovery and, consequently, optimal growth and survival. The rich content of bioactive compounds such as flavonoids and polyphenols in MOLE is likely to increase hemoglobin and red cells, leading to stimulation of the process of erythropoiesis (Rufchaei et al., 2021). This pattern of WBC after diet supplementation is indicative of the development of better capacity of fish to combat infections. These observations were further supported by the work on Nile tilapia, where a rise in WBC count was attributed to the immunomodulatory properties of Moringa’s bioactive components (Nassar et al., 2024). Immune cells such as LYM and EOS count further provided strong evidence of MOLE’s role in building a stronger immune system (Nassar et al., 2024). Rohu fed with 5 g MOLE/diets improved hematological and immunological profiles and positively influenced the histobiochemical indices of Nile tilapia when fed with 2 g MOLE/kg diet (Hamed and El-Sayed, 2019). Conversely, Abdel-Tawwab et al. (2010) reported a significant decrease in MON in Nile tilapia fed various levels of green tea, with the difference in the results being a product of the difference in the leaf materials and their composition. The significant alterations observed in MCV and MCH among fish fed fortified diets suggest that the dietary supplementation influenced erythrocyte characteristics and hemoglobin incorporation within red blood cells. MCV reflects the average size of erythrocytes, while MCH indicates the average amount of hemoglobin contained in each red blood cell. Changes in these indices may therefore reflect modifications in erythropoiesis, red blood cell maturation, or nutrient utilization associated with hematopoietic processes. The observed response could be attributed to the presence of bioactive compounds, vitamins, minerals, and antioxidant constituents in the fortified diets, which may enhance erythrocyte production and improve the physiological status of fish. In contrast, MCHC did not differ significantly among dietary treatments, indicating that although erythrocyte size and total hemoglobin content per cell were affected, the concentration of hemoglobin relative to cell volume remained stable. This suggests that the fortified diets did not alter the hemoglobinization efficiency of erythrocytes but rather influenced red blood cell morphology and hemoglobin accumulation proportionally. Similar findings have been reported in fish fed functional diets containing phytogenic additives, where changes in MCV and MCH were associated with improved hematological status and oxygen-carrying capacity without affecting MCHC (Fazio, 2019; Omitoyin et al., 2023). The maintenance of normal MCHC values further indicates the absence of hematological disorders such as hypochromia or erythrocyte damage, suggesting that the fortified diets supported healthy erythropoietic activity and physiological adaptation in the experimental fish.

The increased MCV values observed in fish fed 0.5–1.0% MOLE, in the absence of significant changes in MCHC, suggest a macrocytic normochromic erythrocyte pattern. This response may reflect enhanced erythropoietic activity and the release of relatively larger immature erythrocytes into circulation, potentially stimulated by the rich antioxidant, vitamin, and bioactive phytochemical content of MOLE. Such compounds have been reported to support hematopoiesis and erythrocyte production through improved nutrient utilization and protection of hematopoietic tissues from oxidative damage (Reverter et al., 2014; Fazio, 2019). Conversely, the reduction in MCV at higher dietary inclusion levels (1.5–2.0% MOLE) may indicate a normalization of erythrocyte size or a shift toward the production of smaller, more mature erythrocytes. This response could be associated with dose-dependent physiological adaptations to higher levels of plant bioactive compounds, including tannins, saponins, or other secondary metabolites, which may influence erythrocyte maturation dynamics and cellular morphology (Reverter et al., 2014). The absence of significant changes in MCHC across treatments suggests that hemoglobin concentration within erythrocytes remained relatively stable, indicating preservation of erythrocyte integrity and oxygen-carrying capacity despite variations in cell size (Reverter et al., 2014).

The incorporation of MOLE into the diets of Heteroclarias, as shown in the results, demonstrated a linear, significant enhancement in antioxidant enzyme activities (GPx, SOD, CAT, and GST) with a concurrently notable reduction in MDA level, indicating decreased lipid peroxidation, especially in fish fed on 2.0 g MOLE/kg diet, which reflects a reduced rate of cellular damage. Studies on Nile tilapia revealed that mole-enriched diets improved oxidative stress resilience (El‐Son et al., 2022). This results further highlights the efficacy of MOLE in enhancing the defense mechanism and reducing the oxidative stress in fish. These antioxidants improve overall fish health by neutralizing reactive oxygen species (ROS) to protect cells from oxidative stress. The antioxidant-boosting role of MOLE in the current study further confirms the protective effects of MOLE in aquaculture feeds​ (Taştan and Salem, 2021; Peñalver et al., 2022; Reda et al., 2023), which are due to its nutritional and antioxidant properties in line with the high content of phenolic compounds. The consistent linear improvements in antioxidant enzyme activities and the concomitant reduction in MDA levels in fish fed MOLE-based diets emphasize the efficacy of MOLE as a natural antioxidant.

Liver enzyme activity of fish provides important information on its health status, especially liver function during supplementation of herbs in fishes (Tiamiyu et al., 2016; Adeshina et al., 2018a; Khalil et al., 2020; Adeniyi et al., 2021; El‐Son et al., 2022; Paray et al., 2024). With the exception of ALT, the activity of AST and ALP remained unchanged across all levels of MOLE. It means that MOLE did not induce any liver damage. Liver function also remained unaffected in stinging catfish, *Heteropneustes fossilis*, when fed with diets containing MOLE (Peñalver et al., 2022). As liver enzymes are markers of hepatic stress, stability in the levels of these markers indicated that the use of moringa in fish diets is safe for prolonged use. AST, ALT, and ALP primarily reflect hepatocellular injury, membrane leakage, or hepatobiliary tissue integrity. These enzymes do not adequately reflect hepatic metabolic or synthetic function. Therefore, this limitation in this study is acknowledged further study.

Assessment of non-specific immune activities such as RBA and LYZ has become a common tool in the evaluation of fish immunity (Adeshina et al., 2018b). LYZ is a component of the innate immune system that plays a crucial role in defense against foreign invasion due to its opsonization properties (Kamble et al., 2024). Studies have shown the stimulatory roles of phytobiotics on RBA and LYZ in fish (Abdel-Tawwab et al., 2010; Hamed and El-Sayed, 2019; El‐Son et al., 2022). In this study, a significant improvement in both LYZ and RBA levels was recorded in fish fed on dietary MOLE, especially those fed with 1.5 to 2.0 g MOLE/kg diet as compared to those fed with the control diet. An increase in lysozyme and respiratory burst activities further confirmed the immunostimulatory effects of MOLE. Immune-boosting properties of plant extracts like MOLE in aquaculture are also revealed by the latest scientific studies. Lysozyme breaks the bacterial cell walls, thus disrupting the activity of bacterial pathogens. Diets enriched with MOLE stimulates the lysozyme activity, leading to the destruction of fish pathogens. A rapid increase in respiratory burst activities further intensifies the activity of lysozyme. These findings suggest that MOLE supplementation can bolster innate immune functions in Heteroclarias. This study agrees with what was observed in Nile tilapia (El-Kassas et al., 2022) and guppy, *Poecilia reticulata* (Secombes and T., 1992), fed with graded dietary MOLE, which demonstrated a higher RBA and LYZ than fish fed on a control diet. Lysozyme activity and respiratory burst activity are functional indicators of innate immune competence, reflecting the antimicrobial capacity and oxidative killing ability of phagocytic cells, respectively. The observed increase in these parameters suggests that dietary MOLE stimulated the activity and efficiency of immune cells, particularly neutrophils and macrophages, rather than increasing their numbers. Differential leukocyte counts provide information on the proportion of circulating leukocyte types but do not measure their activation state or functional performance (Secombes and T., 1992). Therefore, the enhanced lysozyme and respiratory burst activities observed in MOLE-fed fish may indicate improved cellular responsiveness and immune readiness, while the stable lymphocyte and neutrophil percentages suggest that MOLE did not induce leukocyte proliferation or redistribution. This pattern is consistent with previous studies reporting that phytogenic feed additives can enhance innate immune functions by activating existing immune cells and increasing the production of antimicrobial molecules without significantly affecting leukocyte counts (Secombes and T., 1992; Dawood et al., 2018; Fazio, 2019).

Also, to assess immunostimulatory roles of a phytobiotic, a pathogen challenge test is very important. In this study, Heteroclarias were protected against aeromoniasis, and this protection occurred because of increased immunological responses due to the addition of MOLE in fish diets. This immune and antimicrobial activity of MOLE may be associated with those active compounds with antimicrobial properties such as furaneol, maltol, and thiophene, among others. Further, the role and characteristic of essential compounds, including furaneol, maltol and thiophen, are their ability to stimulate hydrophobic actions, which enable them to screen the lipids of the bacterial cell membrane and mitochondria and thus expand the cell structure to render it more permeable and strengthen the overall health of fish (Reverter et al., 2014). Diets enriched with plant-based extracts significantly enhanced the survival rates in fish infected with bacterial pathogens (Adeshina et al., 2018a).

The beneficial effects of dietary MOLE observed in the present study are likely attributable to its rich composition of biologically active phytochemicals, including flavonoids, phenolic acids, tannins, saponins, alkaloids, vitamins, carotenoids, and other antioxidant compounds. These constituents have been widely recognized for their growth-promoting, immunomodulatory, and antioxidant properties in aquatic animals. The enhancement of lysozyme activity and respiratory burst activity in fish fed MOLE indicates stimulation of the innate immune system. Lysozyme serves as an important antimicrobial enzyme capable of hydrolyzing bacterial cell walls, whereas respiratory burst activity reflects the capacity of phagocytic cells to generate reactive oxygen species for pathogen destruction. The improvement of these immune parameters suggests that MOLE may enhance the functional activity of immune cells rather than increasing their abundance. Bioactive compounds such as flavonoids and phenolics are known to modulate immune signaling pathways, stimulate macrophage and neutrophil functions, and promote the production of antimicrobial molecules, thereby strengthening host defense mechanisms (Fazio, 2019).

The antioxidant effects of MOLE may also have contributed significantly to the observed improvements in fish health. Under intensive culture conditions, excessive production of reactive oxygen species can induce oxidative stress, leading to cellular damage, impaired physiological functions, and weakened immunity. The high concentrations of natural antioxidants present in MOLE can neutralize free radicals, reduce lipid peroxidation, and preserve cellular integrity. Consequently, improved antioxidant protection may help maintain normal metabolic processes and support the efficient allocation of energy toward growth and immune functions. The positive effects of MOLE on growth performance may be linked to both nutritional and physiological mechanisms. In addition to supplying essential nutrients, the phytochemicals contained in MOLE may enhance digestive enzyme activity, improve nutrient digestibility, and promote gut health. Improved nutrient utilization increases the availability of energy and metabolic resources required for tissue growth, immune responses, and maintenance functions. This interaction highlights the close relationship between nutrition and immune competence in fish. Furthermore, the improved survival of fish following *A. hydrophila* challenge is likely a consequence of the combined effects of enhanced immune responsiveness and improved oxidative status. A robust innate immune system enables rapid recognition and elimination of invading pathogens, while an effective antioxidant defense system minimizes tissue damage associated with infection-induced oxidative stress. The challenge test demonstrated that dietary supplementation with MOLE enhanced the resistance of fish against bacterial infection, as evidenced by the significantly higher survival rates observed in the supplemented groups compared with the control. The Kaplan–Meier survival analysis further confirmed this protective effect, with supplemented fish exhibiting significantly greater survival probabilities and lower cumulative mortality throughout the challenge period. Notably, fish fed the diet fortified with 1.5 g MOLE kg^-^¹ achieved the highest survival rate (93.3%), whereas mortality occurred earlier and more extensively in the control group, which recorded only 46.5% survival by day 5 post-infection. These findings suggest that dietary MOLE improved the ability of fish to withstand pathogenic stress and mount an effective defense response following infection. The improved post-challenge survival may be attributed to the immunostimulatory and antioxidant properties of *M. oleifera*. The plant is rich in bioactive compounds, including flavonoids, phenolics, vitamins, and carotenoids, which have been reported to enhance innate immune responses and protect host tissues from oxidative damage during infection. This explanation is supported by the increased lysozyme and respiratory burst activities observed in the present study, indicating enhanced antimicrobial capacity and phagocytic function in fish receiving MOLE-supplemented diets. Enhanced innate immune responses can improve pathogen clearance, reduce disease severity, and ultimately increase survival following bacterial challenge (Harikrishnan et al., 2011; Sharker et al., 2024).

Furthermore, the reduced cumulative mortality observed in the supplemented groups may reflect an improved physiological status resulting from the combined effects of enhanced antioxidant defense and immune competence. During bacterial infection, excessive production of reactive oxygen species can damage host tissues and compromise survival (Harikrishnan et al., 2011). The antioxidant constituents of *M. oleifera* may help maintain cellular integrity and mitigate oxidative stress, thereby supporting recovery and resilience during disease outbreaks. Similar improvements in disease resistance and post-challenge survival have been reported in fish fed diets supplemented with phytogenic feed additives rich in antioxidant and immunomodulatory compounds (Harikrishnan et al., 2011). Therefore, the present findings indicate that dietary MOLE, particularly at 1.5 g kg^-^¹, can serve as a functional feed additive for enhancing host defense mechanisms and improving disease resistance in cultured fish.

Therefore, the increased disease resistance observed in MOLE-fed fish appears to result from synergistic interactions among growth enhancement, immune stimulation, and antioxidant protection. Collectively, these findings suggest that MOLE functions as a multifunctional phytogenic feed additive capable of improving fish performance through integrated effects on nutrition, immunity, antioxidant defense, and pathogen resistance. Such properties support its potential application as a sustainable and environmentally friendly alternative to synthetic growth promoters and chemotherapeutic agents in aquaculture.

## 5 Conclusion

The result of this study provides the importance of *Moringa oleifera* leaf extract as a dietary supplement in fish feed. Diets enriched with MOLE have a beneficial role in improving growth performance, blood profile, antioxidant capacity and immune functioning in fish. MOLE has a positive impact on liver functioning, and its inclusion at an optimum rate of 1.35 g MOLE/kg in feed has a very promising effect on the physiology and overall growth of cultured fish. The findings of this study demonstrate that MOLE is a promising natural feed additive for aquaculture. Its inclusion in fish diets can enhance feed utilization efficiency, strengthen antioxidant defenses, improve innate immune responsiveness, and increase resistance to bacterial infections. These multifunctional benefits highlight the potential of MOLE as a sustainable and environmentally friendly dietary supplement that supports fish health, productivity, and welfare. Consequently, MOLE may serve as a viable alternative to synthetic growth promoters and health-management additives, contributing to the development of more resilient and sustainable aquaculture production systems.

## Data Availability

The data that support the findings of this study are openly available in Havard Dataverse at https://doi.org/10.7910/DVN/MLMNPA.
